# Long-Term Outcomes Following Laparoscopic and Abdominal Supracervical Hysterectomies

**DOI:** 10.1155/2010/989127

**Published:** 2010-03-14

**Authors:** Marit Lieng, Annwe Birthe Lømo, Erik Qvigstad

**Affiliations:** Department of Gynecology and Obstetrics, Oslo University Hospital Ullevål, Kirkeveien 166, 0407 Oslo, Norway

## Abstract

Long-term outcomes, in terms of cervical stump symptoms and overall patient satisfaction, were studied in women both after abdominal (SAH) and laparosocopic (LSH) supracervical hysterectomies. Altogether, 134 women had SAH and 315 women LSH during 2004 and 2005 at our department. The response rate of this retrospective study was 79%. Persistent vaginal bleeding after the surgery was reported by 17% in the SAH group and 24% in the LSH group. Regular bleeding was reported by only 8% in both study groups, and the women rarely found the bleeding bothersome. The women reported a significant pain reduction after the surgery, but women having a hysterectomy because of pain and/or endometriosis should be informed about the possibility of persistent symptoms. The overall patient satisfaction after both procedures was high, but the patients should have proper preoperative information about the possibility of cervical stump symptoms after any supracervical hysterectomy.

## 1. Introduction

Hysterectomy is the ultimate treatment for women suffering from symptomatic fibroids, abnormal uterine bleeding and uterine malignancy and is one of the most frequent performed surgical procedures [1, 2]. There is no universal agreement about the optimal method of hysterectomy—abdominal, laparoscopic, or vaginal—and there is a question whether the cervix should be removed as a routine part of the hysterectomy. 

The world's first successful supracervical abdominal hysterectomy (SAH) was performed in 1853 by Gilman Kimball in USA. Since then, the advantages and disadvantages of supracervical versus total hysterectomy technique have been discussed, with variable enthusiasm in different time periods and between countries. More recently, there has been a swing back to supracervical, with marked geographic variations [3–6]. In Scandinavia, the ratio of supracervical to total hysterectomy is traditionally high. At our department in Oslo, Norway, supracervical hysterectomy is the recommended procedure for women with benign conditions requiring hysterectomy and with no previous history of cervical dysplasia. Although laparoscopic supracervical hysterectomy (LSH) has gradually replaced abdominal hysterectomy, SAH is still performed in women where laparoscopic or vaginal approach is not feasible, mainly due to significant enlarged uterus [7].

Opponents of supracervical hysterectomy, either it is performed open or by a laparoscopic approach, often seem to be concerned with the risk of cervical stump symptoms such as vaginal bleeding and pelvic pain following the hysterectomy, causing patient distress and eventually repeated surgery. In a previous publication reporting long-term outcomes after LSH, we found that although vaginal bleeding and pelvic pain are frequently observed following LSH, the patient satisfaction following the procedure was high [8]. 

In this study, we wanted to evaluate whether the occurrence of vaginal bleeding and persistent pelvic pain are consequences of the cervix-sparing technique, or related to the surgical approach. Here we present the long-term outcomes in terms of cervical stump symptoms, women acceptability of such symptoms, and overall patient satisfaction after both abdominal and laparoscopic supracervical hysterectomies performed during the same time period.

## 2. Material and Methods

Following ethical approval, all women who were treated by LSH and SAH on the basis of a benign condition during 2004 and 2005 at the Department of Gynaecology, Oslo University Hospital Ullevål, Oslo, Norway, were sent a questionnaire. Nonresponders were sent a reminder letter four weeks following the original mail out. Firstly, the LSH-treated group were contacted between 12 and 36 months after surgery, while the SAH-treated group were contacted somewhat later, between 17 and 41 months after surgery.

The questionnaire was divided into two sections. The first section contained questions about reasons for having the hysterectomy, as well as menstrual pain and bleeding prior to the hysterectomy. In the second section, questions about experiences of menstrual bleeding and pain following the hysterectomy, any further treatments for such symptoms, any new symptoms related to the hysterectomy and overall satisfaction with the surgery were included. Standard 10-point visual analogue scales (VAS) were used to measure pain intensity and the extent to which bleeding was bothersome. The remaining questions were either dichotomous yes or no responses, or they provided women with either four or five categories of responses to choose from.

Data were analysed using SPSS for Windows (SPSS 14.0, SPSS, Inc, Chicago, IL, USA). Normally distributed continuous data from two groups of women were analysed using a two-sided Independent Samples Student *t*-test and when paired, the Paired Samples *t*-test. Categorical data were analysed using Pearson Chi-Square. Forward stepwise logistic regression analysis was used to calculate the adjusted odds ratios for continued menstrual/cyclical pain, continued vaginal bleeding, and patient satisfaction.

## 3. Results

Altogether, 134 women were identified as having had an SAH and 315 women an LSH during 2004 and 2005 and were therefore sent a questionnaire. Twelve women could not be contacted (five women in SAH group and seven women in LSH group); nine had moved to unknown addresses, and three had died from nongynecological conditions. The response rate in the two groups of women was 82% (SAH group : 106/129) and 78% (LSH group : 240/308), respectively. 

Out of all 449 procedures, 228 (51%) were performed in 2004 and 221 (49%) in 2005. The total response rates for 2004 and 2005 were 75% and 81%, respectively. Mean age of the responders was 48 years (SD 7) in the SAH group and 45 years (SD 6) in the LSH group. There were no significant differences between responders and nonresponders in terms of age and incidence of repeated surgery in either of the two groups of women.

### 3.1. Reasons for Having the Hysterectomy

Most women in both treatment groups (59% in SAH group, 70% in LSH group) stated two or more reasons for having the hysterectomy, the dominating reasons being fibroids (86% versus 68%) and/or heavy bleeding (50% versus 67%). Among women having SAH, 22% stated pain and 4% endometriosis as a reason for the hysterectomy. Respectively, in the LSH group, 46% of women stated pain and 16% endometriosis as a reason for the hysterectomy.

### 3.2. Menstrual Bleeding

Nineteen women (5%) had reached the menopause before the hysterectomy, and three women had medically induced amenorrhoea. Self-reported preoperative menstrual data were available for 96 women in the SAH group and 220 women in the LSH group. The majority in both treatment groups reported their preoperative periodic bleedings to be very heavy (38% in SAH group versus 43% in LSH group) or heavy (18% versus 25%). The remaining women reported their preoperative periodic bleedings as moderately heavy (21% versus 18%), normal (19% versus 11%), or minimal (5% versus 3%).

Out of the responders, 19 women (17%) in the SAH group and 57 women (24%) in the LSH group reported experiencing vaginal bleedings after the hysterectomy. When comparing the occurrence of vaginal bleeding in the two treatment groups, the difference was not statistically significant (*P* > .05). Among women who underwent SAH, ten women (9%) reported to experience regular periodic bleedings, and nine (8%) experienced irregular vaginal bleedings. In the LSH group, 16 women (7%) experienced regular periodic bleedings, 25 women (10%) irregular bleeding patterns, seven women (3%) bleedings in relation to sexual activity, and the remaining nine women (4%) a combination of regular and irregular bleedings which also were related to sexual activity. In the SAH group, no significant age-related differences were found (OR 0.95, 95% CI: 0.87, 1.05), and reason for hysterectomy appeared not to influence the occurrence of persistent vaginal bleeding (Table 1). Adjusted odds ratios (OR) revealed that older women who had been treated by LSH were less likely to experience persistent vaginal bleeding (OR 0.89, 95% CI: 0.83, 0.95). Furthermore, vaginal bleedings after the procedure were less likely to be reported by women who had LSH because of pain (OR 0.41, 95% CI: 0.20, 0.85) or fibroids (OR 0.47, 95% CI: 0.23, 0.93), but more likely to be reported in women with heavy periods prior to surgery (OR 4.07, 95% CI: 1.32, 12.57) (Table 1). 

All women who experienced persistent vaginal bleedings reported the amount of bleeding to be minimal (88% in SAH group, 90% in LSH group), or less than their normal preoperative periodic bleeding (12% versus 10%). The mean degree of bother caused by vaginal bleedings after the hysterectomy, scored on a 10-point VAS, was 1.7 (SD 2.7) in the SAH group and 1.1 (SD 2.0) in the LSH group, respectively.

### 3.3. Pain

Out of the responders, 60 women (55%) in the SAH group and 178 women (74%) in the LSH group suffered from menstrual pain before the hysterectomy. The preoperative mean pain score measured by a 10-point VAS was 5.3 (SD 2.5) in the SAH group and 6.8 (SD 2.1) in the LSH group, respectively. Twenty-three women (21%) in the SAH group and 89 women (37%) in the LSH group reported continued menstrual/periodic pain following their hysterectomy. The mean pain score after hysterectomy was significantly less than before surgery after both procedures (SAH: mean pain score 2.3, SD 1.9, mean pain reduction 2.5, 95% CI: 1.3, 3.7, *P* < .01; LSH: mean pain score 3.5, SD 2.2, mean pain reduction 3.3, 95% CI: 2.7, 3.9, *P* < .01).

Whilst all women reported a significant decrease of pain intensity experienced after the hysterectomy, women having a hysterectomy because of pain and/or endometriosis reported significant higher levels of remaining menstrual/cyclical pain after both procedures (SAH: mean pain score 3.4, SD 2.1; LSH: mean pain score 3.5, SD 2.9), compared with women who did not report endometriosis and/or pain as a reason for the hysterectomy (SAH: mean pain score 1.9, SD 1.7; LSH: mean pain score = 0.9, SD 1.7), *P* = .05 and *P* < .001, respectively (Table 2). The adjusted odds ratios (OR) revealed that increased intensity of preoperative pain resulted in a greater chance of experiencing pain after both procedures (SAH: OR 3.9, 95% CI: 2.0, 5.7; LSH: OR 1.1, 95% CI: 1.0, 1.3). 

### 3.4. Repeated Surgery

In total, 30 out of 449 women (7%) had a further related surgery after the hysterectomy (6% after SAH, 7% after LSH), the most common procedures being performed because of postoperative bleeding, hematomas with secondary infection and adhesions (Table 3). Three of the seven women who went on to have their cervix removed had their original hysterectomy because of endometriosis. Out of the women who reported experiencing continued menstrual bleeding after the hysterectomy, 11% and 7% underwent repeated surgery after SAH and LSH, respectively.

### 3.5. New Symptoms Following Hysterectomy

Out of the responders, 81 women (26% in SAH group, 22% in LSH group) reported experiencing new symptoms following their hysterectomy. Although some women reported to suffer from different forms of pain (pelvic pain, dyspareunia, pain related to the scar) after the hysterectomy, the majority of symptoms reported by the women appeared to be related to the menopause (vasomotoric symptoms, vaginal dryness, reduced libido, gained weight) (Table 4).

#### 3.5.1. Satisfaction with Surgery

Almost all women reported being satisfied (SAH: 28%, LSH: 20%) or very satisfied (SAH: 58%, LSH: 70%) with the hysterectomy. After both procedures, women who reported being satisfied with the preoperative information were more likely to report being very satisfied with the hysterectomy (SAH: OR 5.5, 95% CI: 2.4, 13.0; LSH: OR 3.3, 95% CI: 1.8, 6.2). No significant difference regarding degree of total satisfaction was found comparing women who experienced persistent vaginal bleeding and women who had no vaginal bleeding after the hysterectomy. In both groups, women who reported having a new symptom following their hysterectomy, were less likely to report being very satisfied (SAH: OR 0.2, 95% CI: 0.1, 0.6; LSH: OR 0.2, 95% CI: 0.1, 0.4). No significant difference regarding degree of total satisfaction was found comparing women who had repeated surgery following SAH or not, whereas women who had repeated surgery following LSH were less likely to report being very satisfied (OR 0.1, 95% CI: 0.1, 0.5).

## 4. Discussion

This study reports the occurrence of long-term outcomes following both LSH and SAH in women with benign conditions, as well as the impact these outcomes have on the women's experiences. The relatively large sample size and an excellent response rate represent strengths of the study. Although all procedures were performed at the same department during the same time period, comparisons between the outcomes after the two supracervical procedures should be interpreted with care, as the women were selected and not randomised to the treatment groups. As the benefits of a laparoscopic compared with an abdominal approach are well documented, it would be unethical to randomise women to LSH or SAH [9]. Given these limitations, our study enables a comparison of long-term outcomes after cervix-sparing surgery with both laparoscopic and abdominal techniques. Other limitations of the study are that the data were collected retrospectively and that the questionnaires were sent to the SAH group after the results of the LSH group were known. Furthermore, we were unable to compare the results of supracervical hysterectomy to those of total hysterectomy irrespective of surgical approach.

The results of our study demonstrate that although cervical stump symptoms are relatively common following the two surgical procedures, the overall patient satisfaction is high. The occurrence of vaginal bleedings after SAH is in previous studies reported to be 7%–20% [10–12]. Similarly, the occurrence of persistent vaginal bleedings following LSH is reported in the wide range of 0%–25% [13–17]. The occurrence of vaginal bleedings after both procedures in our study (18% after SAH and 24% after LSH) were relatively high compared to these previous reports. This may partly be explained by different definition of vaginal bleeding in the different studies. We included both regular and irregular bleedings as well as bleeding related to sexual activity, whereas some previous reports have only reported the occurrence of vaginal bleeding on a regular monthly basis. When we included only regular bleedings in our analyses, the proportion decreased to 8% in both study groups. In spite of the high number of women with continued vaginal bleedings, the women rarely found the bleeding to be bothersome, and it did not affect their overall satisfaction.

Insufficient surgical experience and skill, resulting in amputation above the level of the internal cervical ostium, have been suggested as possible causes of the high occurrence of vaginal bleedings following supracervical hysterectomy [13]. Whether other mechanisms, like more meticulous destruction of any remaining endometrium in the spared cervix, could reduce the occurrence of persistent vaginal bleedings after surgery remains to be demonstrated.

The proportion of women who reported suffering from preoperative menstrual pain in our study was relatively high, 74% in the LSH group and 57% in the SAH group. The pain score after hysterectomy was significantly less than before surgery after both procedures, with less pain in the LSH group, possibly related to less adhesion formation after surgery. Although the pelvic pain was reduced after the procedure, a relatively large proportion of women reported continued pain. Some may argue that women with endometriosis and/or pelvic pain would have a more favourable outcome after total hysterectomy compared with a supracervical procedure. However, to our knowledge, no evidence from randomised control trials suggests that total hysterectomy in women with preoperative pain results in greater pain reduction. Furthermore, an eventual effect of a subsequent removal of the cervix following a supracervical procedure due to pain does not appear to have been reported.

The results indicate that women are overall satisfied regarding the outcome following both supracervical procedures. However, as cervical stump symptoms appear to be relatively common, it is important to inform women preoperatively regarding the risk of persistent menstrual bleedings and/or pain. In our study, women who were not satisfied regarding information, reported a significantly lower degree of total patient satisfaction, which illustrates the importance of proper and adequate preoperative information.

## 5. Conclusion

Persistent vaginal bleedings are relatively common following supracervical hysterectomy. The bleedings are, however, reported by women as minimal and rarely bothersome, and the patient satisfaction following both supracervical procedures is high. All women should have proper preoperative information. Women with pelvic pain and/or endometriosis should in addition be informed of the possibility of persistent pelvic pain and the increased risk of repeated surgery following supracervical hysterectomy.

## Figures and Tables

**Table 1:** Adjusted risk estimates for experiencing persistent vaginal bleeding following abdominal and laparoscopic supracervical hysterectomy.

	Reason for hysterectomy		Number of women with vaginal bleeding	Odds ratio (95% CI)
Supracervical abdominal hysterectomy	Fibroids	Yes	16	0.66 (0.16, 2.71)*
		No	3	
	Heavy bleeding	Yes	11	1.28 (0.49, 3.35)*
		No	8	
	Pain and/or endometriosis	Yes	5	1.37 (0.43, 4.32)*
		No	14	

Supracervical laparoscopic hysterectomy	Fibroids	Yes	33	0.47 (0.23, 0.93)*
		No	24	
	Heavy bleeding	Yes	41	4.07 (1.32, 12.57)*
		No	16	
	Pain and/or endometriosis	Yes	23	0.41 (0.20, 0.85)*
		No	34	

*Forward stepwise logistic regression analysis.

**Table 2:** Pain intensity prior to and after surgery by reasons for hysterectomy.

	Reason for hysterectomy	*N*	Mean pain score prior to surgery* (SD)	Mean pain score after surgery (SD)	Mean diff. in pain scores (SD) [95% CI]	*P*-value
Supracervical abdominal hysterectomy	Fibroids	91	5.4 (2.5)	2.3 (1.9)	2.6 (2.8) [1.4, 3.8]	<.01**
	Heavy bleeding	52	5.6 (2.6)	2.3 (2.0)	2.5 (3.2) [0.7, 4.3]	<.01**
	Endometriosis/pain	27	6.7 (1.9)	3.4 (2.1)	3.0 (3.1) [0.1, 5.9]	.04**

Supracervical laparoscopic hysterectomy	Fibroids	151	4.8 (3.4)	1.1 (1.9)	3.7 (3.3) [3.2, 4.2]	<.001**
	Heavy bleeding	151	5.6 (3.2)	1.3 (2.0)	4.4 (3.4) [3.2, 4.9]	<.001**
	Endometriosis	37	7.5 (2.5)	3.5 (2.9)	4.0 (4.0) [2.7, 5.3]	<.001**
	Pain	104	7.1 (2.6)	1.9 (2.5)	5.3 (3.5) [4.6, 6.0]	<.001*

*10-point VAS,**Paired Samples *t*-test.

**Table 3:** Surgical procedures performed after the hysterectomy.

Surgical procedures after hysterectomy	Supracervical abdominal hysterectomy N*	Supracervical laparoscopic hysterectomy N**
Resurgery within 24 hours due to bleeding	3	—
Laparoscopic adhesiolysis	—	6
Laparoscopic extirpation of cervix uteri	—	7
Drainage of postoperative hematoma/abscess	2	1
Bowel resection due to postoperative peritonitis	1	1
BSOE and removal of cervix uteri (sarcoma uteri)	—	1
Scar correction	1	3
Umbilical hernia repair	—	1
Tension-free vaginal tape procedure (TVT)	—	2
Cystoscopy	1	—

**Total**	**8 (6%)**	**22 (7%)**

*Total number of women having supracervical abdominal hysterectomy: 134.

**Total number of women having supracervical laparoscopic hysterectomy: 315.

**Table 4:** New symptoms reported following the hysterectomy.

New symptoms	Supracervical abdominal hysterectomy *N* (%)	Supracervical laparoscopic hysterectomy *N* (%)
Changes related to urination and/or defecation	4 (3.8)	9 (3.8)
Vasomotor symptoms	—	5 (2.1)
Vaginal discharge	—	5 (2.1)
Vaginal dryness and/or dyspareunia	3 (2.8)	5 (2.1)
Pelvic pain	3 (2.8)	5 (2.1)
Problems related to the scar (pain and/or cosmetics)	6 (5.7)	—
Other (depression, gained weight, reduced libido,	12 (9.0)	24 (10.0)
cystitis, candida infection, fear of cervical cancer)		

## References

[1] Baggish MS (2005). Total and subtotal abdominal hysterectomy. *Best Practice and Research: Clinical Obstetrics and Gynaecology*.

[2] Clayton RD (2006). Hysterectomy. *Best Practice and Research: Clinical Obstetrics and Gynaecology*.

[3] Gimbel H, Settnes A, Tabor A (2001). Hysterectomy on benign indication in Denmark 1988–1998: a register based trend analysis. *Acta Obstetricia et Gynecologica Scandinavica*.

[4] Farquhar CM, Steiner CA (2002). Hysterectomy rates in the United States 1990–1997. *Obstetrics and Gynecology*.

[5] Merrill RM (2008). Hysterectomy surveillance in the United States, 1997 through 2005. *Medical Science Monitor*.

[6] Gimbel H, Bonnar J, Dunlop W (2005). Total or subtotal hysterectomy: what is the evidence. *Recent Advances in Obstetrics and Gyneacology*.

[7] Istre O, Langebrekke A, Qvigstad E (2007). Changing hysterectomy technique from open abdominal to laparoscopic: new trend in Oslo, Norway. *Journal of Minimally Invasive Gynecology*.

[8] Lieng M, Qvigstad E, Istre O, Langebrekke A, Ballard K (2008). Long-term outcomes following laparoscopic supracervical hysterectomy. *British Journal of Obstetrics and Gynaecology*.

[9] Johnson N, Barlow D, Lethaby A, Tavender E, Curr E, Garry R (2006). Surgical approach to hysterectomy for benign gynaecological disease. *Cochrane Database of Systematic Reviews*.

[10] Gimbel H, Zobbe V, Andersen BM, Filtenborg T, Gluud C, Tabor A (2003). Randomised controlled trial of total compared with subtotal hysterectomy with one-year follow up results. *British Journal of Obstetrics and Gynaecology*.

[11] Thakar R, Ayers S, Clarkson P, Stanton S, Manyonda I (2002). Outcomes after total versus subtotal abdominal hysterectomy. *New England Journal of Medicine*.

[12] Ewies AAA, Olah KSJ (2000). Subtotal abdominal hysterectomy: a surgical advance or a backward step?. *British Journal of Obstetrics and Gynaecology*.

[13] Donnez J, Nisolle M, Smets M, Polet R, Bassil S (1997). Laparoscopic supracervical (subtotal) hysterectomy: a first series of 500 cases. *Gynaecological Endoscopy*.

[14] Okaro EO, Jones KD, Sutton C (2001). Long term outcome following laparoscopic supracervical hysterectomy. *British Journal of Obstetrics and Gynaecology*.

[15] Zupi E, Zullo F, Marconi D (2003). Hysteroscopic endometrial resection versus laparoscopic supracervical hysterectomy for menorrhagia: a prospective randomized trial. *American Journal of Obstetrics and Gynecology*.

[16] van der Stege JG, van Beek JJ (1999). Problems related to the cervical stump at follow-up in laparoscopic supracervical hysterectomy. *Journal of the Society of Laparoendoscopic Surgeons*.

[17] Ghomi A, Hantes J, Lotze EC (2005). Incidence of cyclical bleeding after laparoscopic supracervical hysterectomy. *Journal of Minimally Invasive Gynecology*.

